# Research on plant knowledge graph reasoning based on dual-channel attention and topological perception

**DOI:** 10.3389/fpls.2026.1814740

**Published:** 2026-05-08

**Authors:** Shasha Wang, Yongye Su, Hui Gao

**Affiliations:** 1 Virtual Simulation and Big Data Engineering Technology Research Center, School of Data Science, Hebi Polytechnic, Hebi, China; 2 School of Computer Science and Engineering, University of Electronic Science and Technology, Chengdu, China

**Keywords:** attention mechanism, graph neural networks, knowledge graph reasoning, long-range dependency modeling, plant knowledge graph

## Abstract

Knowledge graphs (KGs) in the plant domain frequently contain “long-range dependency” paths (e.g., taxonomic hierarchies or ecological association chains of 4 or more hops), which pose significant challenges to existing KG reasoning models. Traditional Graph Neural Network (GNN) models struggle to effectively capture such long-range dependencies due to issues of long-distance information compression and over-smoothing. To address this, we propose the KRGAI-PLANT model, an inductive reasoning framework specifically designed for plant knowledge graphs. This model features a dual-channel architecture that integrates a global attention mechanism with local topology perception, enabling synergistic learning between global semantic interactions and local structural features among plant entities. We conduct experimental validation on subsets constructed from mainstream plant knowledge graphs, including DBpedia and PlantNet-KG. The results demonstrate that KRGAI-PLANT achieves significant improvements over baseline models such as GraIL and NBFNet in key evaluation metrics, including Hits@10 and AUC-PR, particularly exhibiting strong advantages in handling long-path reasoning tasks. This study provides an effective reasoning tool for knowledge discovery and association prediction in the plant domain. Furthermore, the proposed KRGAI-PLANT model offers a robust cognitive framework that can empower distributed and autonomous agricultural systems by converting complex, multi-source plant data into actionable knowledge for precise decision-making.

## 1 Introduction

Botany, as a data-intensive foundational discipline, features a highly structured and networked knowledge system. With the rapid development of fields such as plant taxonomy, ecology, and genomics, plant-related data have experienced explosive growth. To effectively integrate and utilize these vast amounts of heterogeneous and multi-source data, plant knowledge graphs have emerged and are becoming a core infrastructure for plant intelligent information services Smith et al. (2022). Plant knowledge graphs systematically organize plant entities (e.g., species, genes, compounds, ecological niches) and their semantic relationships (e.g., classification, symbiosis, metabolism, interaction) in a graph structure, providing a unified knowledge base for the digital management of plant resources, knowledge discovery, and intelligent decision-making Wang et al. (2021).This foundational work is crucial for the next generation of distributed and autonomous intelligent systems in crop production. By accurately modeling long-range dependencies and complex relationships within plant ecosystems, such knowledge graphs serve as the essential “digital twin” or reasoning engine, enabling systems to achieve a thorough and detailed understanding of crop status—a core objective of this Research Topic.

Within plant knowledge graphs, entities are not only connected by direct associations but are more extensively linked by deep, complex relationships that require traversal through multiple intermediate nodes and relational chains to reveal—termed “long-range dependency” relationships (typically referring to 4 or more hops) Li et al. (2020). These long-range dependency paths reflect the complex principles of the plant world. For instance, in taxonomy, the chain “rice (Oryza sativa) → genus Oryza → family Poaceae → order Poales → clade Commelinids” constitutes a 5-hop taxonomic hierarchy. In ecology, the chain “Masson’s pine (Pinus massoniana) → infected by → pinewood nematode (Bursaphelenchus xylophilus) → vector → Japanese pine sawyer (Monochamus alternatus) → natural enemy → woodpecker (Picidae)” forms a 5-hop ecological association chain revealing pest transmission and natural enemy control. In medicinal botany, the chain “Chinese yew (Taxus chinensis) → produces → paclitaxel → targets → tubulin → used to treat → cancer” constitutes a 4-hop application value chain. Mining and reasoning such long-range dependency relationships are of paramount importance for advancing research in plant systematics and evolution, ecosystem stability assessment, plant pest and disease transmission path prediction, and medicinal plant resource discovery Li et al. (2018).

However, existing knowledge graph reasoning methods, particularly state-of-the-art inductive reasoning models based on Graph Neural Networks (GNNs), face significant challenges when dealing with long-range dependencies in plant knowledge graphs. Traditional GNNs based on message-passing mechanisms (e.g., Graph Convolutional Networks (GCNs) and Graph Attention Networks (GATs)) suffer from inherent limitations: (1) Long-distance information dilution: when information propagates through multiple hops via successive neighbor nodes, the original signal attenuates severely, making it difficult for the model to perceive the influence of distant entities effectively; (2) Over-smoothing: as the number of network layers increases to capture a larger receptive field, node features across the graph tend to become similar, losing distinctive local structural information and consequently impairing reasoning performance Chen et al. (2020). Consequently, these models excel at capturing local patterns within 1–2 hops but often underperform in long-range reasoning tasks that span multiple biological hierarchies or functional modules.

To address these issues, we propose the KRGAI-PLANT model, aimed at systematically solving the long-range relationship reasoning challenge in plant knowledge graphs. The model comprises two main modules that enhance reasoning capability from both local topology and global semantics perspectives. The Local Topology Structure Module employs a shallow GNN to focus on capturing the neighborhood structural features of nodes. The Global Information Module establishes direct interaction channels across graph nodes via an innovative attention mechanism, overcoming the limitations of traditional GNN message-passing and enabling semantic relationship modeling across the entire graph. The two modules are co-optimized through parameter sharing and feature fusion mechanisms, preserving the spatial constraints of the graph structure while enhancing the model’s capacity to model long-range dependencies.

The main contributions of this paper are as follows:

1. Proposal of a domain-specific model: We introduce the dual-channel reasoning framework integrating global information perception and local topology modeling to the field of botany for the first time, constructing the KRGAI-PLANT model optimized for plant knowledge graphs.
2. Design of an adaptive fusion mechanism: The model innovatively employs an attention-guided feature fusion module, enabling adaptive integration of global semantic information and local structural features tailored to different types of plant entities (e.g., taxonomic groups, functional groups) for more accurate reasoning.
3. Validation in real-world scenarios: We construct high-quality evaluation datasets from large-scale plant knowledge graphs (e.g., DBpedia Plant, PlantNet-KG) and, through comprehensive experiments, demonstrate that KRGAI-PLANT significantly outperforms state-of-the-art baseline models such as GraIL, NBFNet, and RED-GNN across multiple metrics, particularly excelling in long-path reasoning tasks.
4. Preliminary interpretability analysis: Through a case study on a representative ecological chain (Masson’s pine → woodpecker), we visualize the model’s attention weights to illustrate how it captures long-range dependencies. While this provides some intuition, a systematic interpretability evaluation is left for future work.

The structure of the subsequent sections is as follows: Section 2 reviews related work. Section 3 details the overall architecture and key technical details of the KRGAI-PLANT model. Section 4 describes the experimental setup, datasets, baseline models, and provides an in-depth analysis of the results. Section 5 concludes the paper and outlines future research directions.

## 2 Related works

### 2.1 Construction and application of plant knowledge graphs

Plant science is a quintessential data-intensive field, featuring a wide array of highly heterogeneous data types, including taxonomic, morphological, ecological, genomic, and metabolomic information Lowe et al. (2017). To enable the integration of these multi-source datasets for computable knowledge discovery, plant knowledge graphs (KGs) have emerged as a pivotal solution. By systematically organizing plant entities and their semantic relationships into graph structures, these KGs serve as a foundational infrastructure for intelligent plant information services Wang et al. (2021). Existing efforts can be broadly classified into two categories: general-purpose integrated KGs, such as PlantNet-KG Joly et al. (2016) and KNApSAcK Afendi et al. (2012), which aggregate large-scale, cross-domain botanical data; and task-specific thematic KGs, including those designed for crop breeding Zeng et al. (2022) and plant disease diagnosis Chen et al. (2021). Current applications predominantly focus on enhanced knowledge retrieval, interactive visualization, and preliminary association discovery Zhao et al. (2021). However, these approaches largely depend on explicitly encoded knowledge or straightforward graph traversal, failing to fully exploit the complex, implicit logic—such as multi-factor interactions or long-range, cross-hierarchical dependencies—inherent in plant systems. This limitation constrains the potential for deep cognitive analysis and predictive modeling of complex botanical phenomena.

### 2.2 Knowledge graph reasoning methods

Knowledge graph reasoning aims to infer missing relationships or discover novel associations within a graph. Mainstream methodologies fall into two paradigms: symbolic logic-based approaches, such as Neural LP Yang et al. (2017) and RuleN Meilicke et al. (2018), and representation learning (embedding)-based approaches. In recent years, Graph Neural Networks (GNNs) have become the dominant paradigm for relational reasoning due to their superior capacity to model graph-structured data Wu et al. (2020). Nonetheless, classical GNNs, which operate on local message-passing mechanisms, face fundamental challenges in multi-hop reasoning tasks: (1) Long-distance information dilution, where meaningful signals attenuate over extended paths Li et al. (2018); (2) Over-smoothing, where deepening networks cause node representations to homogenize, eroding discriminative structural information Chen et al. (2020). Although recent advances have introduced models like NBFNet Zhu et al. (2021) and RED-GNN Zhang and Yao (2022) to mitigate these issues, offering improved performance in path-based reasoning, these models are predominantly designed for general-purpose use. Consequently, their architectures and optimization objectives are not specifically tailored to address the unique data characteristics and reasoning demands of vertical domain knowledge graphs, such as those in botany.

Comparison with Hybrid GNN-Transformer Architectures. Recently, several hybrid models have attempted to combine GNNs with Transformers for graph-based reasoning. For instance, Graphormer Ying et al. (2021) incorporates structural encodings into Transformer attention, while SGFormer Wu et al. (2023) simplifies the architecture for scalability. However, these models are designed for general graph tasks and lack mechanisms tailored to knowledge graph reasoning, particularly for long-range dependencies in domain-specific KGs. In contrast, KRGAI-PLANT introduces three distinctive design choices: (1) A dual-channel design that preserves local topological integrity while enabling global semantic interaction; (2) An adaptive attention fusion mechanism that dynamically balances local and global information based on entity type—which is particularly relevant for plant KGs where taxonomic entities and ecological niches have heterogeneous information needs; (3) Linear attention to improve scalability to large-scale plant KGs. These design choices aim to better address the unique challenges of plant KG reasoning, including long-range taxonomic chains, sparse ecological relations, and inductive reasoning over unseen species.

### 2.3 Challenges and research gaps in plant knowledge graph reasoning

The inherent complexity of botanical knowledge systems presents three core challenges for automated reasoning over plant KGs:

1. Long-range and cross-subgraph dependencies: Critical biological associations—such as taxonomic lineages, ecological interaction networks, and metabolic pathways—frequently manifest as chains of four or more hops, demanding models capable of capturing long-distance semantic dependencies Li et al. (2020).
2. Extreme heterogeneity and sparsity of relationship types: Plant KGs integrate diverse semantic relations (e.g., taxonomic, ecological, chemical, geographical) with highly imbalanced distributions. Effective models must, therefore, precisely discern and learn from these varied relational patterns.
3. Need for inductive reasoning over unseen entities: The continuous discovery of new species, gene sequences, and interactions necessitates that practical systems perform reliable reasoning on completely new nodes not encountered during training, requiring strong inductive learning capabilities Teru et al. (2020).

Currently, applying generic KG reasoning models directly to the plant domain reveals significant shortcomings. Existing models show limited efficacy in capturing complex associations that span distant biological hierarchies or functional modules and lack robust mechanisms to adapt to the dynamic expansion of plant graphs (e.g., with new entities and relationships). Therefore, there is a pressing need for a novel reasoning framework that can synergistically leverage local topological structure information and global semantic context, explicitly optimized for the long-range dependency, relational heterogeneity, and inductive requirements characteristic of plant knowledge graphs. The KRGAI-PLANT model proposed in this paper is designed to address this research gap.

## 3 The KRGAI-PLANT model

### 3.1 Overall framework

**Figure 1:**
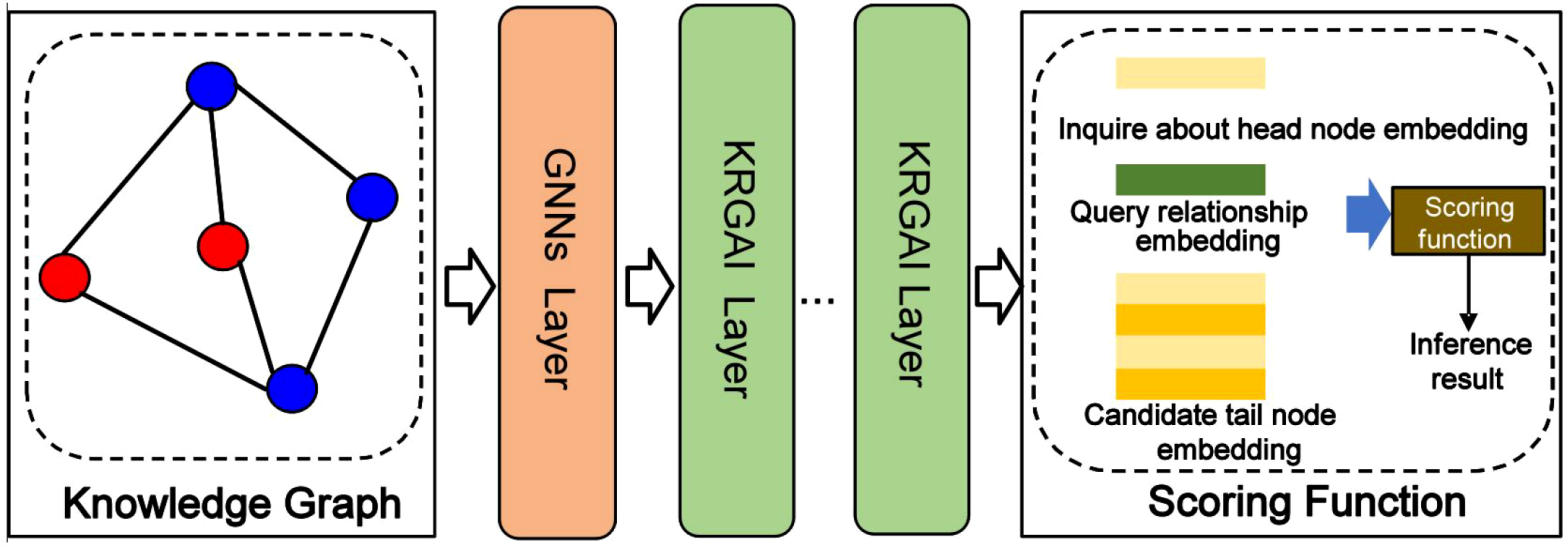
The KRGAI-PLANT model architecture diagram: first, acquiring initial structural features via the base GNN, followed by parallel global-local information interaction within the KRGAI layers.

The overall architecture of the KRGAI-PLANT model is illustrated in **Figure 1**. The model takes a knowledge graph and a reasoning query as input. It first generates initial embedding representations for nodes while loading pre-trained relation embeddings. These embeddings undergo preliminary feature extraction via a base Graph Neural Network (GNN). Subsequently, they are fed into multiple stacked KRGAI layers for deep feature learning. Each KRGAI layer contains two parallel branches: a global attention mechanism and a local topology modeling component, enabling synergistic learning between global semantic information and local structural features. Finally, the model computes scores for candidate tail entities via a scoring function and outputs the reasoning result.

As KRGAI-PLANT automatically generates initial node embeddings, feeding these directly into the global attention module would not benefit the reasoning task. Inspired by the LERP model, we designed a two-stage feature learning process: first, acquiring initial structural features via the base GNN, followed by parallel global-local information interaction within the KRGAI layers. The experimental section will investigate the impact of the number of KRGA layers on model performance given a fixed total depth, providing empirical guidance for model depth configuration.

### 3.2 Single-layer network structure

**Figure 2:**
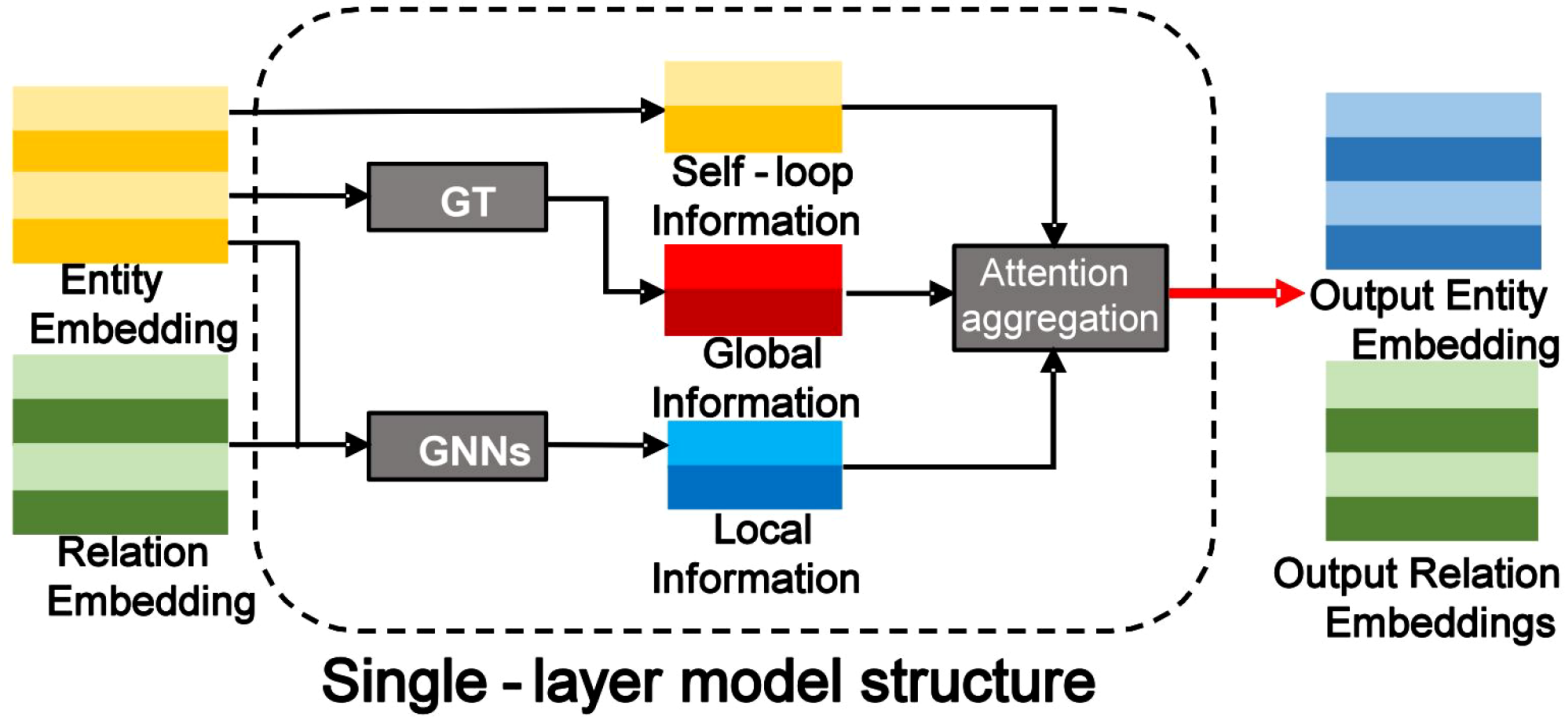
The single-layer structure of the KRGAI-PLANT model: it consists the global information module and the local topology structure module.

**Figure 2** depicts the structure of a single KRGAI layer, which consists of two main components: the Global Information Module and the Local Topology Structure Module. The global module is implemented as a Graph Transformer (GT), while the local module employs a shallow GNN to extract the topological structure around entities. An attention-based aggregation module fuses three features: the global features obtained from the GT, the local features from the GNN, and the entity’s own features treated as a self-loop.

Let the input knowledge graph be denoted as  G=(E,R,T),the E denote the set of entities in the knowledge graph, the R denote the set of relations in the knowledge graph, and T denote the set of triples in the knowledge graph. A triple (h,r,t)∈Tindicates that there exists a relation h∈R between the head entity h∈E and the tail entity h∈E. The operations of the global and local modules for layer l can be formulated as:

(3‐1) $$E_{global}^{\left(l\right)}=\text{GT}(E^{\left(l\right)})$$

(3‐2) $$E_{local}^{\left(l\right)}=\text{GNN}s(E^{\left(l\right)},R^{\left(l\right)},G)$$

In **Equations 3-1** and **3-2**, $E^{\left(l\right)}\in {\mathbb{R}}^{|E|\times d}$, $R^{\left(l\right)}\in {\mathbb{R}}^{|R|\times d}$ are the input entity and relation feature matrices from the previous layer, $E_{global}^{\left(l\right)}$represents the global features obtained by the Global Information Module, $E_{local}^{\left(l\right)}$denotes the local features acquired by the Local Topology Structure Module. Following the acquisition of outputs from both modules, feature fusion is performed. The corresponding formulation is:

(3‐3) $$e_{u}^{\left(l+1\right)}=\alpha_{1}e_{u,0}^{\left(l\right)}+\alpha_{2}e_{u,1}^{\left(l\right)}+\alpha_{3}e_{u,2}^{\left(l\right)}$$

In Equation 3-3, $e_{u}^{\left(l+1\right)}\in {\mathbb{R}}^{d}$denotes the updated entity embedding representation of node *u*, $e_{u,0}^{\left(l\right)},e_{u,1}^{\left(l\right)},e_{u,2}^{\left(l\right)}\in R^{d}$respectively represent the input embedding vector of node *u* retrieved from the embedding matrix, the global feature vector and the local feature vector, $\alpha_{1},\alpha_{2},\alpha_{3}$ respectively denote the weight coefficient vectors computed by the attention mechanism, these coefficients are derived from the following Equations 3-4 and 3-5.

(3‐4) $$\alpha_{i}=\frac{f(w^{\left(l\right)},e_{u,i}^{\left(l\right)})}{\sum_{j=1}^{3}f(w^{\left(l\right)},e_{u,j}^{\left(l\right)})}$$

(3‐5) $$f(w^{\left(l\right)},e_{u,j}^{\left(l\right)})=\exp(\sigma (w^{(l)⊤}e_{u,j}^{\left(l\right)}))$$

Where $w^{\left(l\right)}\in {\mathbb{R}}^{d}$denotes the attention weight vector at the *l*-th layer, σ(·)represents the activation function, typically implemented as the LeakyReLU function. Through this attention mechanism, the model can adaptively select the most relevant features for each node, thereby optimally balancing the importance of three distinct information sources for the current node: the self-loop information, the global information, and the local topological information. For relation features, a linear layer is employed to update the relation embeddings, i.e. $R^{\left(l+1\right)}=W^{\left(l\right)}R^{\left(l\right)}$, or alternatively, layer-independent relation embedding vectors may be used.

#### 3.2.1 Global information module

**Figure 3:**
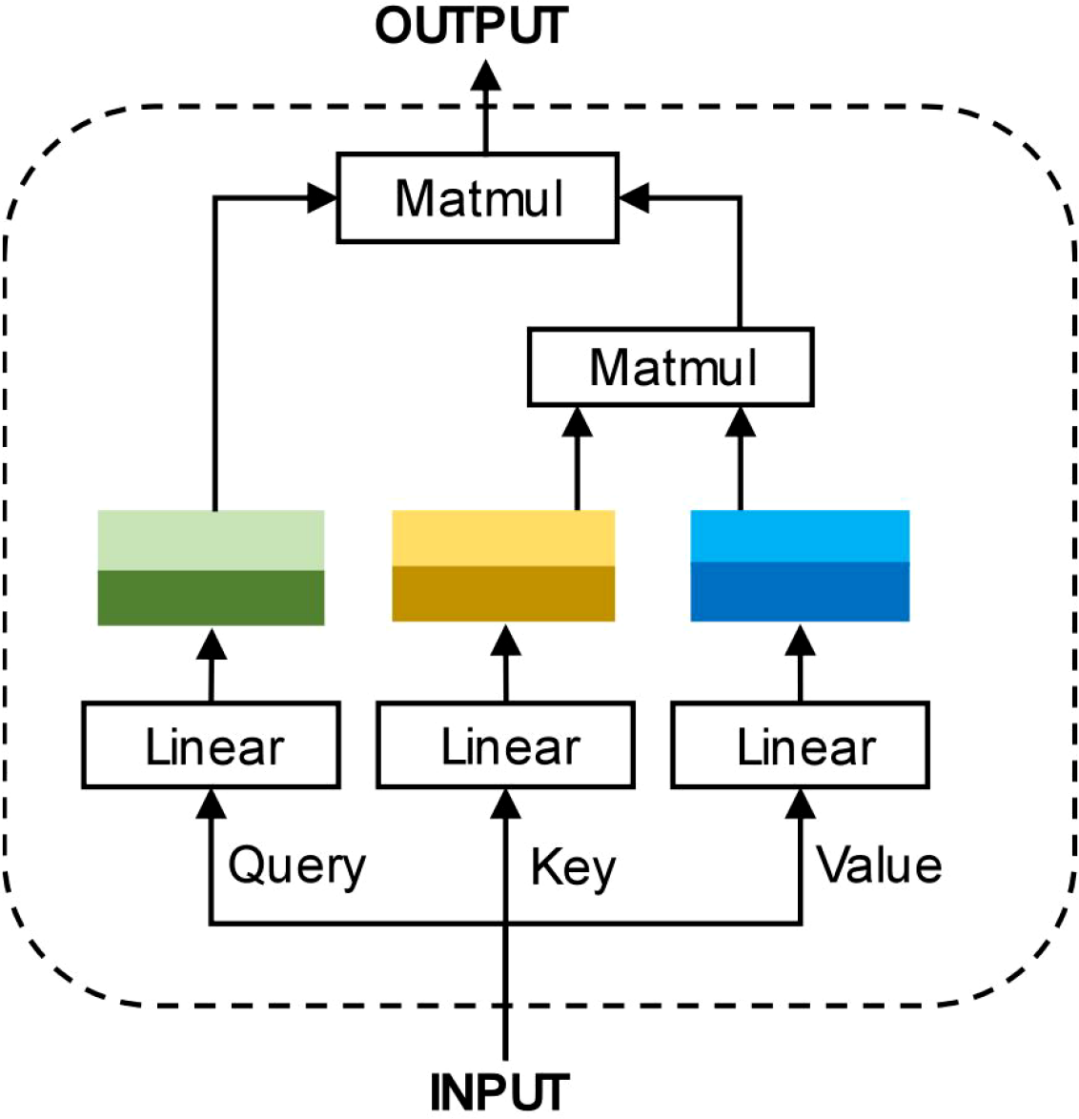
Schematic diagram of the attention mechanism: this module is implemented using a graph transformer architecture, which establishes direct connectivity channels across all nodes in the graph via a self-attention mechanism, enabling any pair of nodes to interact across layers.

As previously mentioned, the long-distance information compression problem in GNNs makes it difficult for nodes to obtain information from distant neighbors, thereby hindering GNNs’ ability to handle knowledge reasoning tasks with long-range dependencies. To address this limitation, the KRGAI-PLANT model introduces a Global Information Module to facilitate global information interaction among nodes. This module is implemented using a Graph Transformer architecture, which establishes direct connectivity channels across all nodes in the graph via a self-attention mechanism, enabling any pair of nodes to interact across layers. The structural diagram of this module is illustrated in **Figure 3**.

Although the attention mechanism employed in the Transformer model Vaswani et al. (2017) enables effective global information interaction, its quadratic time complexity of $O(|\epsilon {|}^{2}d^{\left(l\right)})$renders it impractical for large-scale knowledge graphs. To address this, KRGAI-PLANT adopts a linear attention mechanism, proposed by Katharopoulos et al. (2020), which approximates the standard attention computation using a kernel function ϕ(·). As illustrated in **Figure 3**, the global information module processes node embeddings via this linear attention. Specifically, the input entity embeddings are projected through three separate linear layers to generate the Query(Q), Key(K) and Value (V) matrices. The computation proceeds as follows: first, the Q and K matrices are transformed via the kernel function *ϕ*(·). The transformed K matrix is then multiplied with the V matrix, and the result is subsequently multiplied with the transformed Q matrix to produce the globally informed node representations. Furthermore, KRGAI-PLANT incorporates a multi-head attention mechanism into the global module, allowing the model to attend to diverse feature aspects. Assuming *k* independent attention heads operate in parallel, their computations are formulated in Equations 3-6 and 3-7:

(3‐6) $$Q=W_{Q,h}E^{\left(l\right)},K=W_{K,h}E^{\left(l\right)},V=W_{V,h}E^{\left(l\right)}$$

(3‐7) $$E_{global,h}^{\left(l+1\right)}=\phi (Q)(\phi (W{)}^{⊤}V)$$

Where $Q,K,V\in {\mathbb{R}}^{|E|\times d^{\left(l+1\right)}}$represent the Query, Key and Value matrices, **E**^(^*^l^*^)^ denotes the input entity embedding representation to the module. $E_{global,h}^{\left(l+1\right)}$ denotes the global information feature matrix computed by the *k*-th attention head, $W_{Q,h},W_{K,h},W_{V,h}\in {\mathbb{R}}^{d^{\left(l\right)}\times d^{\left(l+1\right)}}$respectively represent the learnable linear transformation matrices for the Query, Key, and Value channels, and *ϕ*(·) denotes the kernel function. It is typically defined as ϕ(·)=ELU(·)+1.

**Figure 4:**
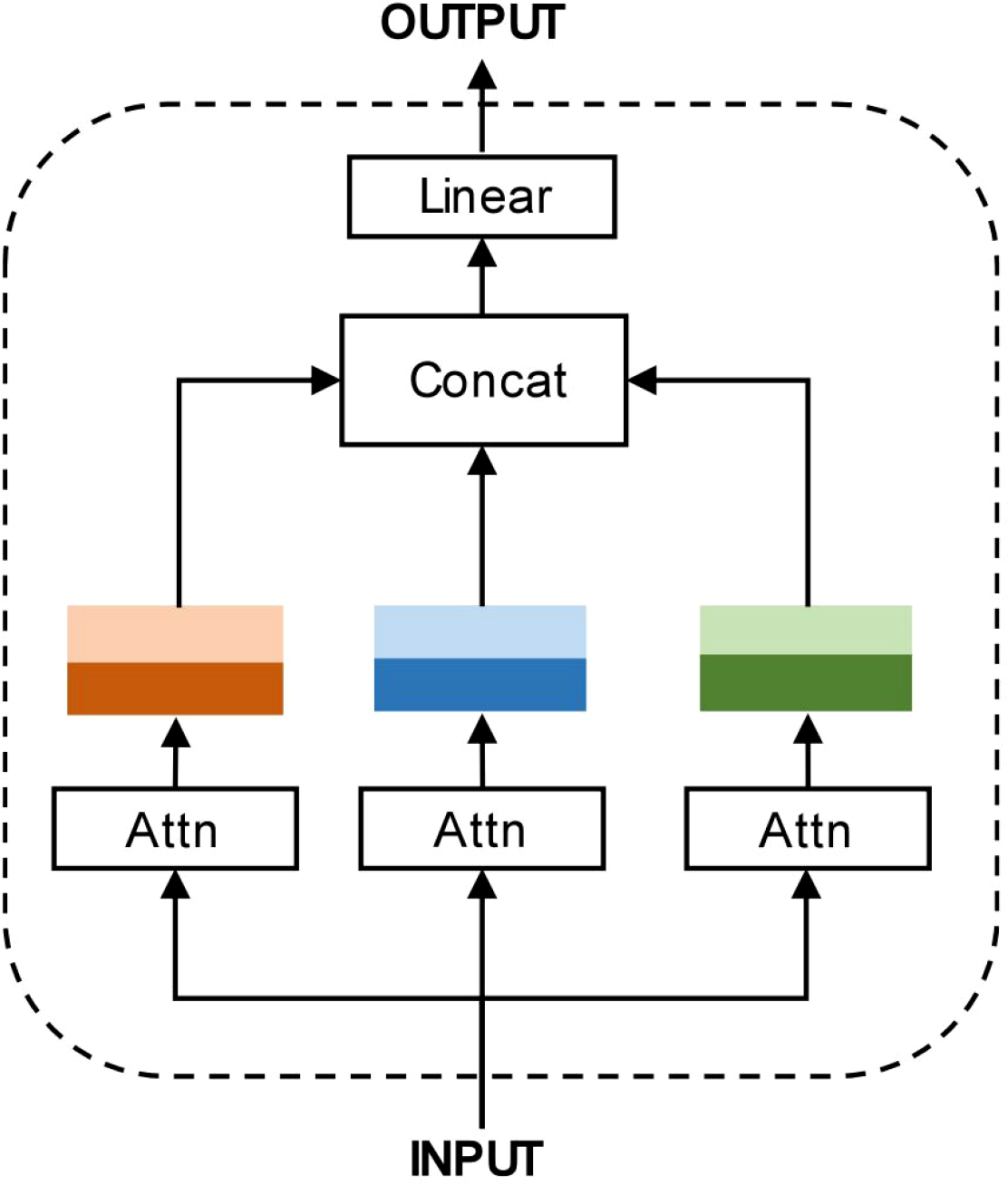
Schematic Diagram of the Multi-Head Attention Mechanism: Once the computations of all attention heads are completed, the information from each head is fused via concatenation.

As shown in **Figure 4**, once the computations of all attention heads are completed, the information from each head is fused via concatenation. This process is formulated in Equation 3-8:

(3‐8) $$E_{global}^{\left(l+1\right)}=\text{concat}(E_{global,1}^{\left(l+1\right)},E_{global,2}^{\left(l+1\right)},\ldots ,E_{global,k}^{\left(l+1\right)})$$

Where $E_{global}^{\left(l+1\right)}$denotes the updated embedding representation from the Global Information Module, $W_{m}\in {\mathbb{R}}^{d^{\left(l\right)}\times d^{\left(l+1\right)}}$denotes the linear transformation matrix applied after the multi-head attention fusion, concat(·) denotes the vector concatenation operation.

#### 3.2.2 Local topology structure module

Inspired by the positional encoding mechanism in the Transformer model, we recognize that the local neighborhood structure of nodes in a knowledge graph contains crucial semantic information and plays a key role in reasoning tasks. Therefore, the KRGAI-PLANT model employs a shallow GNN within the Local Topology Structure Module to capture neighborhood features through a limited number of message-passing iterations. This process is formulated in Equation 3-9:

(3‐9) $$e_{u}^{\left(l+1\right)}={\sum_{\left(r,v)\in N(u\right)}\beta }_{\left(u,r,v\right)}^{\left(l\right)}\phi (u,r,v)$$

Where $e_{u}^{\left(l+1\right)}\in {\mathbb{R}}^{d^{\left(l+1\right)}}$denotes the updated local topological structure feature of node u, N(u)represents the first-order neighborhood of node *u*, (r,v)∈N(u) indicates that node *v* is connected to node *u* via relation *r*,*ϕ(u,r, v)* is the message generation function that integrates features from node *u*,node *v* and relation *r* to produce a message passed from node *v* to node *u*, $\beta_{\left(u,r,v\right)}^{\left(l\right)}$signifies the attention weight assigned by node *u* to node *v*, calculated according to the following **Equations 3-10** and **3-11**:

(3‐10) $$\gamma_{\left(u,r,v\right)}^{\left(l\right)}=\sigma (W_{att}^{\left(l\right)}\phi (u,r,v))$$

(3‐11) $$\beta_{\left(u,r,v\right)}^{\left(l\right)}=\frac{\exp(\gamma_{\left(u,r,v\right)}^{\left(l\right)})}{\sum_{\left(i,j)\in N(u\right)}\exp(\gamma_{\left(u,i,j\right)}^{\left(l\right)})}$$

Where $W_{att}^{\left(l\right)}\in {\mathbb{R}}^{1\times d^{\left(l+1\right)}}$denotes the attention linear transformation matrix of the Local Topology Structure Module at the *l*-th layer.

### 3.3 Scoring function and loss function

After obtaining the entity embedding matrix **E** and relation embedding matrix **R** through the multi-layer KRGAI-PLANT model, as shown in **Equation 3-12**, for a candidate triple *(h,q,t)*, its features are concatenated and fed into a two-layer Perceptron(MLP) with hidden dimension 64. The hidden layer uses ReLU activation, and the output layer uses sigmoid activation to produce a probability score between 0 and 1. This scoring function can be formulated as:

(3‐12) $$p(h,q,t)=sigmoid(MLP(e_{h}\|r_{q}\|e_{t}))$$

The model is trained using a negative sampling strategy. As shown in **Equation 3-13**, for each positive triple (h,q,t), a corresponding negative triple (h,q,t′) is constructed by randomly replacing the tail entity within the graph. Both positive and negative samples are fed into the model simultaneously, with the objective of maximizing the score for positive triples while minimizing it for negative triples. Since the MLP outputs a probability value, the loss function can be defined as:

(3‐13) $$L=-\log p(h,q,t)-\frac{1}{n}\sum_{i=1}^{n}\log(1-p(h,q,t'))$$

**Figure 5:**
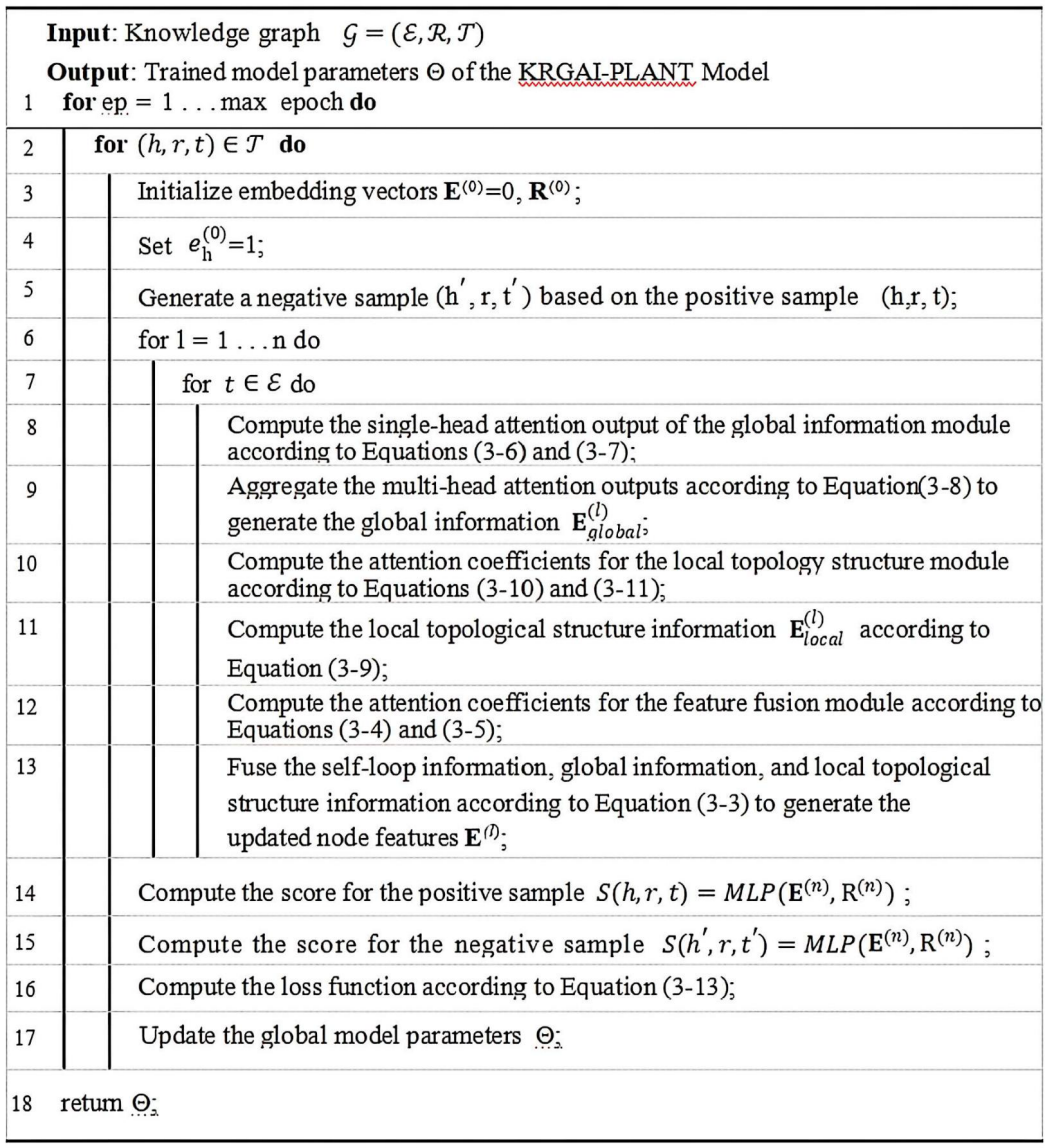
Training procedure of the KRGAI-PLANT model.

The detailed training procedure of the KRGAI-PLANT model is outlined in **Figure 5**.

## 4 Experimental results and analysis

This section systematically evaluates the performance of the KRGAI-PLANT model using a standardized experimental protocol and evaluation framework. First, we introduce the statistical properties and task settings of two widely adopted datasets, including the number of entities, relation types, and the splits for training, validation, and testing. Next, we detail the experimental hyperparameter configurations (e.g., learning rate, batch size, embedding dimension) and the selection of baseline models for comparison. Subsequently, based on standard evaluation metrics, we report the performance of KRGAI-PLANT on knowledge reasoning tasks and conduct comparative analyses against the baselines. Finally, we design ablation studies to analyze the contribution of each module and investigate the impact of key hyperparameters on model performance, thereby comprehensively validating the effectiveness and robustness of the KRGAI-PLANT model.

### 4.1 Datasets

To evaluate the inductive reasoning capability of KRGAI-PLANT on plant knowledge graphs, we constructed two dedicated botanical datasets: PN-KG and DPS. Both datasets were built from publicly available sources and processed through a systematic pipeline consisting of three stages: (1) raw data acquisition, (2) entity/relation filtering and normalization, and (3) inductive subgraph partitioning.

PlantNet Focused Knowledge Graph (PN-KG): PN-KG is a domain-specific, highly structured, multilingual knowledge graph constructed for the plant domain. It covers over 200,000 plant species and contains multi-hop taxonomic relations (e.g., species → subspecies → variety → genus → family, 5 hops), ecological associations (e.g., plant → habitat → soil type → climate zone → geographic region, 5 hops), and morphological feature chains (e.g., plant → leaf shape → leaf venation → leaf margin → plant organ → morphological classification, 5 hops). Examples of long-range dependencies include a 5-hop taxonomic path: sunflower (species) → variety → oil sunflower → genus Helianthus → family Asteraceae → order Asterales; and a 6-hop ecological path: orchid → epiphyte → host tree → growth environment → tropical rainforest → climate type → tropical monsoon climate.

DBpedia Plant Subset (DPS): This dataset is a high-quality, structured subset specifically extracted from the general DBpedia knowledge graph, focusing on plants. Its core data is built from Wikipedia’s structured information. It includes botanical taxonomic hierarchies (species → genus → family → order → class → phylum → kingdom, up to 6–7 hops), morphological features (e.g., “leaf shape → leaf venation type → plant organ”), and ecological associations (e.g., “growth environment → climate zone → geographic region”). Examples of long-range dependencies include a 5-hop taxonomic path: a plant species → belongs to → a genus → belongs to → a family → belongs to → an order → belongs to → a class; and a 5-hop ecological association path: a plant → companion plant → another plant → pest/disease → natural enemy insect → belonging order.

PN-KG was constructed by querying the PlantNet Knowledge Graph API (https://plantnet.org/open-data/). We retrieved entities and relations for over 200,000 plant species, focusing on taxonomic, ecological, and morphological relationships. The raw JSON responses were parsed into triplets, followed by entity deduplication (merging entities with identical scientific names), relation type normalization (mapping diverse relation labels to a standardized set of 9–11 core types), and removal of low-confidence entries (confidence score < 0.8).

DPS was extracted from the DBpedia SPARQL endpoint (https://dbpedia.org/sparql) by selecting entities with rdf:type dbo:Plant. We retained taxonomic relations (dbo:family, dbo:genus, dbo:order, dbo:class, dbo:phylum, dbo:kingdom), habitat associations (dbo:habitat), and morphological attributes. Only entities with at least three hierarchical levels in their taxonomic path were retained to ensure sufficient structural complexity for long-range dependency evaluation. Relations appearing fewer than five times were removed to reduce noise.

Inductive Subgraph Partitioning. Following the inductive learning protocol established by GraIL (Teru et al., 2020), we partitioned each complete graph into four disjoint subgraph versions (v1–v4) using a random walk-based graph sampling algorithm. The sampling parameters were set as follows: walk length = 50, walks per node = 10, restart probability = 0.3. The sampled subgraphs were then randomly assigned to training (75% of subgraphs) and test (25% of subgraphs) sets, ensuring that test graphs contain entirely unseen entities not appearing in the training graphs. The partitioning was repeated with different random seeds (42, 123, 456, 789) to generate four independent versions. The dataset statistics are summarized in **Table 1**, and the average path distances are reported in **Table 2**.

**Table 1:** Statistics of the evaluation datasets.

Dataset		DPS			PN-KG		
		Relation num	Entity num	Triple num	Relation num	Entity num	Triple num
V1	Training set Test set	89 75	2000 1500	5226 2404	99 45	2746 900	6678 1900
V2	Training set Test set	76 59	3000 2000	12085 5092	96 73	6954 2900	18968 4800
V3	Training set Test set	69 84	4000 3000	22394 9137	101 82	12078 5000	32150 7500
V4	Training set Test set	46 82	5000 3500	33916 14554	74 87	3861 7200	9842 15000

**Table 2:** Average distance of all reachable node pairs.

Dataset	DPS		PN-KG	
	Training set	Test set	Training set	Test set
v1	7.68	5.6	3.83	5.2
v2	7.34	5.1	3.82	6.3
v3	8.02	4.7	3.63	6.8
v4	7.21	4.4	3.58	5.5

In addition to the basic statistics of the test datasets, **Table 2** presents the average distance of all reachable node pairs for each version of the DPS and PN-KG datasets. The distance between a pair of nodes is computed by converting each directed edge into an undirected edge with a weight of 1.

### 4.2 Evaluation metrics and parameter settings

Following the setup of the GraIL model Teru et al. (2020), we employ AUC-PR (Area Under the Precision-Recall Curve) and Hits@10 as the core evaluation metrics. AUC-PR provides a comprehensive assessment of the precision-recall trade-off across varying thresholds, effectively reflecting the overall reasoning performance of the model under highly imbalanced class distributions. Hits@10 measures the proportion of correct entities appearing within the top-10 ranked predictions, focusing on the ranking accuracy of the model in specific reasoning queries.

Regarding the training parameters, for the KRGAI-PLANT model configuration, the embedding dimension is fixed at 32 for all models. The total number of model layers is selected from {1, 2, 3, 4, 5, 6}. For each positive sample, the number of generated negative samples is chosen from {32, 64, 128, 256, 512, 1024}. In terms of optimizer settings, we adopt the Adam optimizer, with the learning rate tuned among {10^-^², 10^-3^, 10^-4^, 10^-5^}. The weight decay coefficient for L2 regularization is selected from {10^-4^, 10^-5^, 10^-6^, 10^-7^}. Finally, for the training strategy, the number of positive samples per batch is chosen from {16, 32, 64}, and all models are trained for a total of 20 epochs.

All experiments are conducted on a physical server equipped with an Intel(R) Xeon(R) Gold 5220 CPU @ 2.20GHz processor and an NVIDIA A800 80GBGPU, using the PyTorch framework for training.

### 4.3 Time complexity analysis

This section analyzes the time complexity of each module in the KRGAI−PLANT model.

1. For the Local Topology Structure Module, which employs GNNs based on the message−passing and aggregation mechanism, the time complexity is $O(|T|d+|\epsilon |d^{2})$, here |T|d accounts for the message passing and aggregation steps, and $|\epsilon |d^{2}$corresponds to the linear transformation of features, where denotes the embedding dimension.
2. For the Global Information Module, which replaces the softmax function with a kernel−based approach, the time complexity for a single attention head is $O(2d^{2}|\epsilon |)$,Assuming the module uses attention heads, the overall complexity becomes $O(nd^{2}|\epsilon |)$.

In summary, if the KRGAI−PLANT model is stacked with layers, the total time complexity is $O(Ld|T|+Lnd^{2}|\epsilon |)$

### 4.4 Baseline models

During the experimental validation phase, we selected several models as baselines for comparative analysis. These models cover both rule-based reasoning models and graph neural network-based reasoning models, as detailed below:

1. GraIL Teru et al. (2020): An inductive graph reasoning model that performs relational inference through subgraph extraction and graph attention mechanisms. It pioneered a local enclosed subgraph sampling strategy, effectively handles compositional relations in knowledge graphs, demonstrates strong generalization in few-shot scenarios, and is well-suited for dynamic graph environments.
2. Neural LP Yang et al. (2017): A neural logic programming model that transforms symbolic reasoning into differentiable tensor operations. It employs neural tensor networks to learn soft logical rules, supports end-to-end training, balances rule interpretability with model performance, and is suitable for handling complex multi-hop reasoning tasks.
3. RuleN Meilicke et al. (2018): A rule induction-based reasoning system that generates inference rules by mining closed path patterns. It utilizes an efficient path search algorithm, achieves high recall with low computational resources, and is applicable to rule discovery in few-shot knowledge graphs.
4. DRUM Sadeghian et al. (2019): A dual-channel rule model that combines symbolic rules with embedding representations. Through dynamic rule instantiation and embedding refinement mechanisms, it enhances rule scalability and can handle sparse relation reasoning in large-scale knowledge graphs.
5. NBFNet Zhu et al. (2021): A message-passing-based path reasoning model that learns generalized path features via a generalized Bellman-Ford algorithm. It overcomes the depth limitations of traditional GNNs, supports reasoning over arbitrary hops, and excels in modeling long-range dependencies.
6. RED-GNN Zhang and Yao (2022): A relation-enhanced diffusion graph neural network model that introduces a relation-aware neighborhood diffusion mechanism. By controlling the information propagation scope through adaptive diffusion coefficients, it effectively mitigates over-smoothing and enhances representation learning in multi-relational graphs.
7. AstarNet Zhu et al. (2023): A heuristic graph search reasoning model that integrates the A* algorithm with neural networks. It employs a learnable heuristic function to guide reasoning path generation, improving search efficiency while maintaining the interpretability of the reasoning process.
8. AdaProp Zhang et al. (2023): An adaptive propagation model whose core is based on meta-learning or attention mechanisms. It adaptively aggregates neighborhood information according to relation types and path contexts, enhancing modeling capability for sparse or complex relations and performing notably well in multi-hop reasoning tasks.
9. InGram Lee et al. (2023): An inductive graph representation learning model specifically designed for few-shot reasoning over new entities. It constructs a cross-graph meta-representation space via prototype networks, supports zero-shot relation inference, and shows significant advantages in dynamic open-domain scenarios.

In summary, these baselines represent three paradigms for KG reasoning: rule-based (Neural LP, RuleN, DRUM), path-based (NBFNet, RED-GNN, AstarNet), and subgraph-based (GraIL, AdaProp, InGram). KRGAI-PLANT differs by combining global attention with local topology through an adaptive fusion mechanism specifically designed for plant KGs.

### 4.5 Experimental results

**Table 3** presents the experimental results for the Hits@10 metric on the DPS and PN-KG datasets, while **Table 4** shows the results for the AUC-PR metric on the same two datasets. In these tables, the best-performing results are highlighted in bold, and the second-best results are marked with an underline.

**Table 3:** Experimental results of hits@10 on test datasets, the best-performing results are highlighted in bold, and the second-best results are marked with an underline.

Models	DPS				PN-KG			
	v1	v2	v3	v4	v1	v2	v3	v4
GraIL	69.3	72.1	48.3	62.2	47.4	40.0	38.4	36.1
Neural LP	70.5	69.6	46.6	71.0	40.8	55.2	51.8	54.9
RuleN	66.6	60.6	38.1	64.6	38.8	52.4	52.4	50.1
DRUM	70.9	67.4	40.7	66.6	41.3	56.1	51.8	54.3
NBFNet	75.4	73.0	53.2	65.9	52.8	65.2	54.6	60.3
RED-GNN	72.9	70.4	52.0	68.4	42.1	59.3	45.7	57.1
AstarNet	73.9	71.4	50.9	70.5	51.3	**66.3**	57.9	59.5
Adaprop	73.6	**74.9**	**55.0**	**71.6**	50.1	65.8	52.2	52.8
InGram	55.3	43.3	19.9	24.6	42.9	45.4	12.9	37.7
KRGAI-PLANT	**76.5**	73.3	52.7	70.6	**54.0**	**67.0**	**62.5**	**60.7**

The best-performing results are highlighted in bold, and the second-best results are marked with an underline.

**Table 4:** Experimental results of AUC-PR on test datasets, the best-performing results are highlighted in bold, and the second-best results are marked with an underline.

Models	DPS				PN-KG			
	v1	v2	v3	v4	v1	v2	v3	v4
GraIL	88.41	89.71	79.39	86.93	82.25	87.59	90.08	91.16
Neural LP	79.74	77.32	69.29	76.70	72.62	77.58	70.49	74.14
RuleN	85.59	82.18	76.58	82.34	74.25	86.02	85.59	88.69
DRUM	82.59	80.59	63.08	80.01	76.32	7523	76.29	73.64
NBFNet	93.28	94.05	**93.21**	**95.09**	91.87	93.82	94.08	90.53
KRGAI-PLANT	**93.57**	**94.26**	90.31	92.06	**92.39**	**94.35**	**95.85**	**95.03**

The best-performing results are highlighted in bold, and the second-best results are marked with an underline.

As shown in **Tables 3**, **4**, the KRGAI-PLANT model demonstrates significant advantages across all eight datasets in terms of both Hits@10 and AUC-PR metrics. Under the Hits@10 metric, KRGAI-PLANT achieves the best performance on all four versions of PN-KG (v1–v4), and ranks first on DPS-v1. On DPS-v2 and DPS-v4, it attains second-best performance, trailing the top-performing model (AdaProp) by margins of 1.3 and 1.0 points, respectively. On DPS-v3, KRGAI-PLANT ranks third, behind AdaProp and NBFNet. This performance distribution indicates that the advantage of KRGAI-PLANT is most pronounced on datasets with longer average reasoning paths, such as PN-KG. In terms of AUC-PR, KRGAI-PLANT achieves the best performance on six datasets: DPS-v1/v2 and PN-KG-v1/v2/v3/v4, while securing the second-best position on the remaining two datasets (DPS-v3/v4). Notably, in the v1 and v4 versions of PN-KG, KRGAI-PLANT improves the Hits@10 score by 1.4 and 1.3 points, respectively, over the second-best model. This margin underscores the advantage of the KRGAI-PLANT model in inductive reasoning over knowledge graphs.

The experimental results indicate that the KRGAI-PLANT model performs better across the four versions of PN-KG than it does across the four versions of DPS. This performance disparity is closely related to the inherent characteristics of the two dataset types. As shown in **Table 2**, there is a significant difference in the average distance of reachable node pairs between the various versions of DPS and PN-KG, reflecting distinct differences in relational path lengths and structural complexity between the two datasets. This difference stems from the distinct construction strategies of the two datasets. PN-KG was built by tracing complete ecological and taxonomic chains from the PlantNet database (e.g., orchid → epiphyte → host tree → tropical rainforest climate), which naturally produces longer paths. In contrast, DPS test sets were sampled to preserve local density within specific taxonomic groups (e.g., all species within a single family or genus), resulting in shorter average distances while maintaining high intra-group connectivity. Consequently, PN-KG provides a more challenging testbed for evaluating long-range reasoning capabilities, which explains why KRGAI-PLANT’s advantage is most pronounced on this dataset.

On the DPS dataset, a notable discrepancy exists between the training and test sets regarding the average distance of reachable node pairs. As shown in **Table 2**, the training set exhibits long average path lengths (7.21–8.02 hops), encompassing rich cross-hierarchical associations spanning multiple taxonomic levels. In contrast, the test sets show significantly shorter average paths (4.4–5.6 hops), likely because their construction emphasizes sampling highly interconnected entities within specific taxonomic groups (e.g., a particular family or genus).

On the PN-KG dataset, the average distance of reachable node pairs in the training set ranges from 3.58 to 3.83 hops, while in the test sets, this average distance increases to between 5.2 and 6.8 hops. This increase—particularly evident in PN-KG-v3 (6.8 hops)—indicates a substantially higher demand for long-range dependency modeling capability in the test tasks.

Limitations and failure cases. Despite its overall superior performance, KRGAI-PLANT is not universally better across all scenarios. In our experiments, we observed that on DPS-v3, KRGAI-PLANT ranked third behind AdaProp and NBFNet (**Table 3**). This dataset version has the highest density among DPS test sets (average degree 6.09), suggesting that when local graph structure alone provides sufficient information, the additional global attention mechanism may offer diminishing returns. Furthermore, queries requiring more than 8 hops (rare in our test sets) may challenge the linear attention’s ability to propagate signals over extremely long distances. Future work could investigate adaptive mechanisms that selectively activate the global module based on query characteristics.

### 4.6 Ablation studies and analysis

This section conducts ablation studies across a total of 8 datasets (from DPS and PN-KG) to validate the impact of the Global Information Module and the Local Topology Structure Module on model performance. The design details of the ablation experiments are as follows:

1. Removal of the Global Information Module: In the single-layer KRGAI-PLANT structure, the KRGAI-PLANT structure, the global feature vector $e_{u,1}^{1}$computed by the Graph Transformer (GT) is removed. Furthermore, in Equations 3-4 and 3-5, the corresponding coefficient is set to zero to obtain the updated embedding representation.
2. Removal of the Local Topology Structure Module: In the single-layer KRGAI-PLANT structure, the local feature vector $e_{u,2}^{1}$computed by the GNNs is removed. Additionally, in Equations 3-4 and 3-5, the corresponding coefficient α_3_ is set to zero to derive the updated embedding representation.

**Figure 6:**
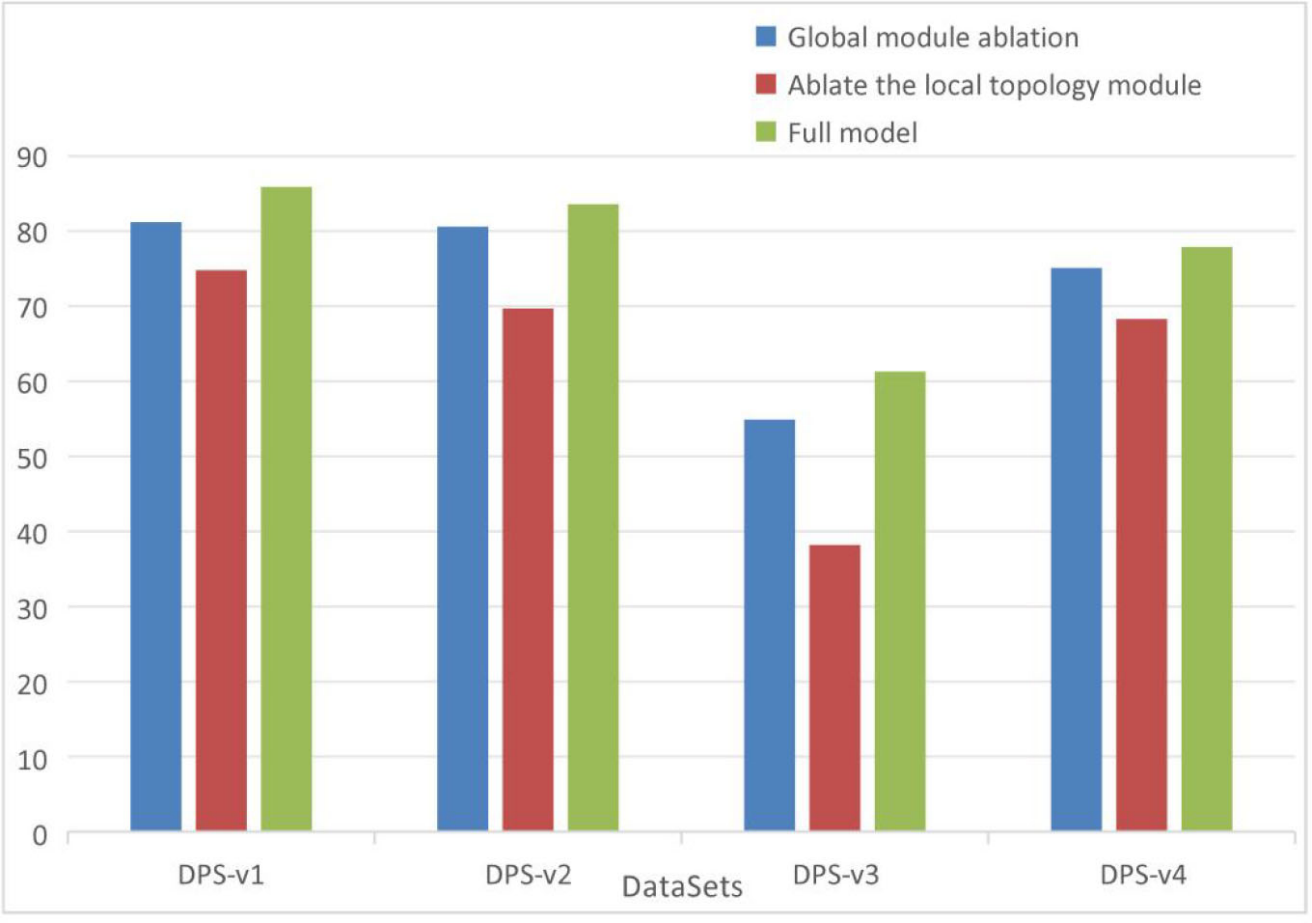
Ablation study results on the DPS dataset.

**Figure 7:**
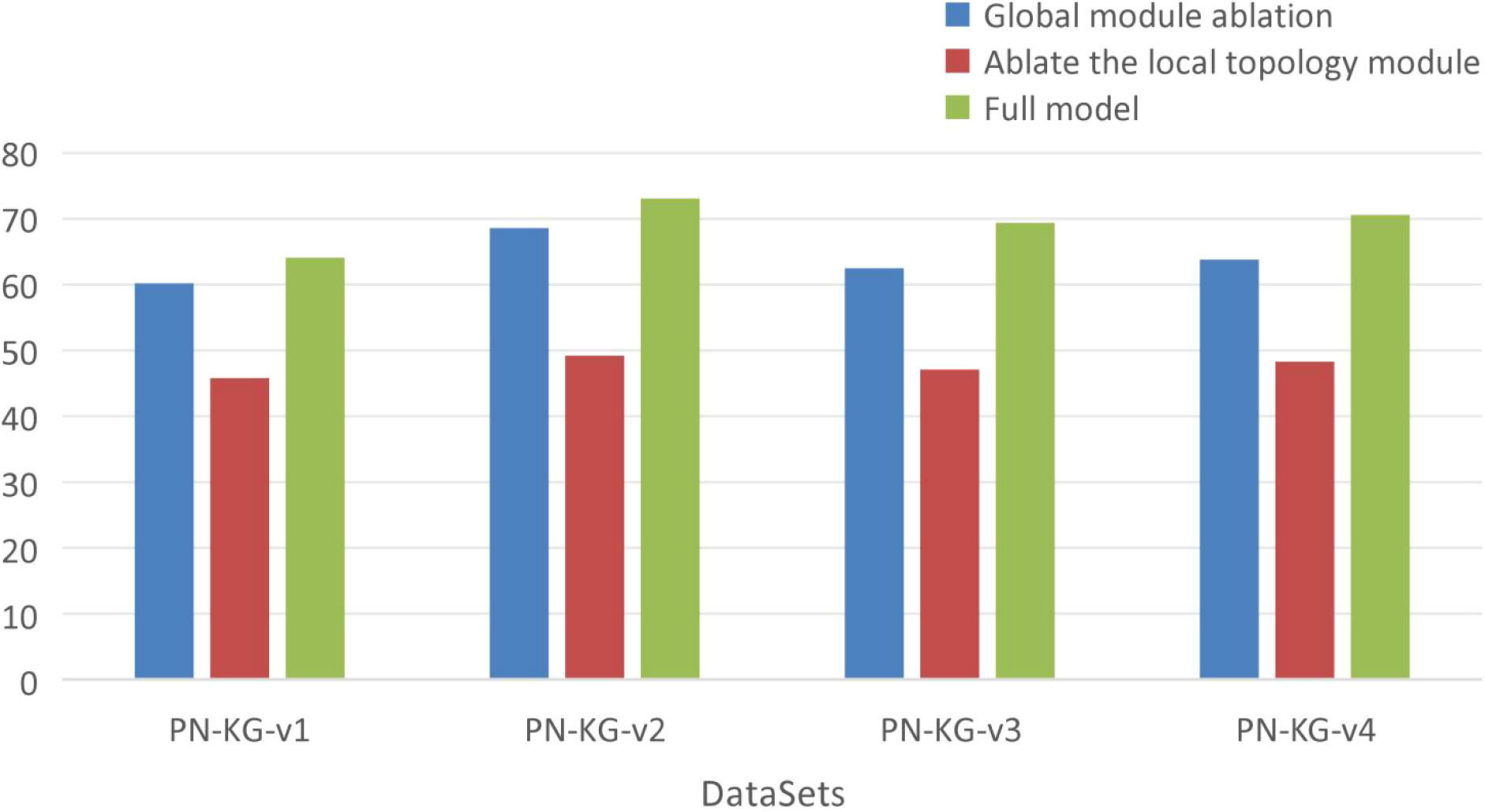
Ablation study results on the PN-KG dataset.

The results of the ablation experiments based on the Hits@10 metric are presented in **Table 5**, with corresponding bar charts shown in **Figures 6**, **7**.

**Table 5:** Impact of the local topology structure module and the global information module on model performance: the results of the ablation experiments based on the Hits@10 metric.

Model	DPS				PN-KG			
	v1	v2	v3	v4	v1	v2	v3	v4
Global module ablation	81.2	80.6	54.9	75.1	60.2	68.6	62.5	63.8
Ablate local topology module	74.8	69.7	38.2	68.3	45.8	49.2	47.1	48.3
Full model	85.9	83.6	61.3	77.9	64.1	73.1	69.4	70.6

As shown in **Table 5**, after separately removing the Global Information Module and the Local Topology Structure Module, the performance of the KRGAI-PLANT model exhibits varying degrees of degradation across different datasets. This indicates that both the Global Information Module and the Local Topology Module play critical roles in the KRGAI-PLANT model’s performance across diverse datasets.

From a quantitative perspective, it is evident that removing the Local Topology Structure Module leads to a drastic decline in the performance of the KRGAI-PLANT model. This can be attributed to two main factors:

1. The core of knowledge graph reasoning lies in modeling semantic associations based on graph structures. Deviating from this structural foundation inevitably severely impacts performance. Once the Local Topology Structure Module is removed, the KRGAI-PLANT model loses the ability to perceive the topological information around nodes. It can only infer semantic associations between two entities through the scoring function, which critically undermines its capability to handle graph-structured data.
2. The Global Information Module does not incorporate interactions between relations and node features. In the full model, the GNN within the Local Topology Module dynamically fuses node features and relation semantics through message passing. Removing this module decouples the update processes of entity embeddings and relation embeddings, thereby weakening the model’s expressive power for complex relations.

The Local Topology Module serves as the structural cornerstone for knowledge graph reasoning. Its role extends beyond capturing neighborhood features; it fundamentally constructs a dynamic interaction space between entities and relations. In contrast, the Global Module functions as a feature enhancement component primarily addressing long-range dependencies. Both modules are indispensable. However, removing the Local Module directly undermines the foundational logic of graph data modeling, which explains why the performance degradation is particularly severe. As visualized in **Figures 6**, **7**, the performance drop is particularly pronounced when removing the local module, confirming its foundational role.

### 4.7 Hyperparameter experiments and analysis

This section conducts experimental analyses on several key hyperparameters of the KRGAI-PLANT model to examine how different choices affect its performance. The hyperparameter experiments performed include: (1) the impact of the total number of model layers on performance; (2) the influence of the initial number of GNN layers under a fixed total model depth; (3) the effect of different methods for fusing local structural features with global information features.

#### 4.7.1 Impact of model depth (number of layers) on performance

To investigate how model depth influences knowledge reasoning capability, we designed a systematic experiment evaluating the performance of the KRGAI-PLANT model with varying numbers of stacked layers across multiple datasets. By incrementally increasing the number of KRGAI-PLANT layers, we analyze the relationship between network depth and task adaptability.

**Figure 8:**
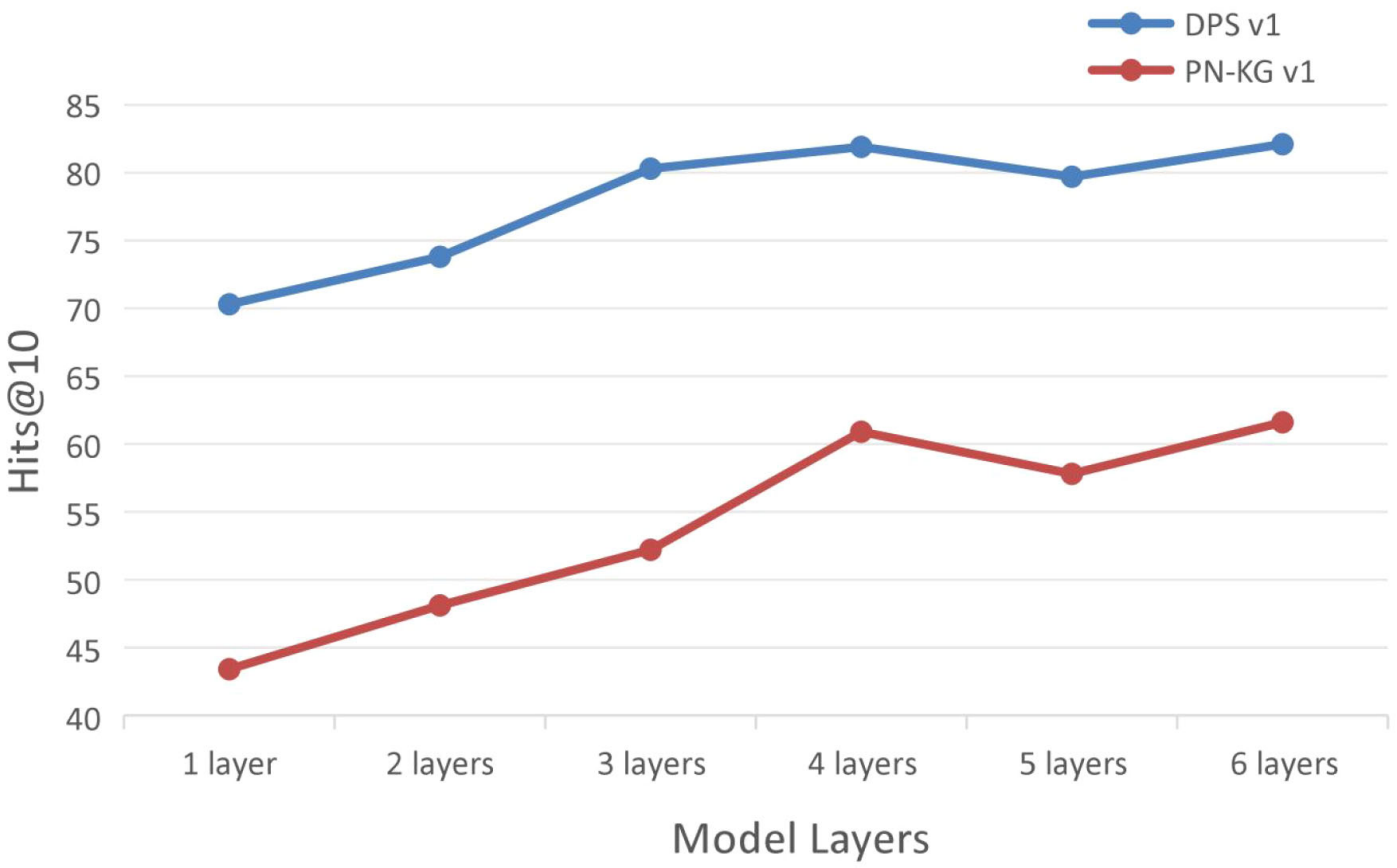
Impact of model depth (number of layers) on performance.

The analysis based on **Figure 8** leads to the following observations:

1. As the number of model layers increases, overall model performance generally improves, indicating that deeper networks enhance the feature extraction capabilities of both the Local Topology Structure Module and the Global Information Module.
2. Model performance exhibits a significant dataset-dependent correlation with depth. Specifically, on the DPS dataset, the Hits@10 metric peaks at 4 layers and then slightly declines due to over-smoothing. In contrast, on the PN-KG dataset, performance dips briefly at 5 layers before rising again to its highest value at 6 layers. This discrepancy can be explained by the structural characteristics of the datasets. Referring to the average reachable pair distances in **Table 2**, DPS has shorter average distances, causing the Local Topology Structure Module to be more prone to over-smoothing, which diminishes modeling capacity. In contrast, PN-KG’s longer average distances allow the KRGAI-PLANT model to maintain the effectiveness of the local module while simultaneously leveraging the Global Information Module to effectively capture long-range dependencies.

#### 4.7.2 Impact of the initial number of GNN layers on model performance

To validate the effectiveness of the two-stage feature learning mechanism, we designed a controlled experiment. Under the constraint of a fixed total network depth of 6 layers, we systematically evaluate the impact of the allocation between the number of initial GNN layers and the number of KRGAI-PLANT layers on model performance. Specifically, by incrementally increasing the number of initial GNN layers while correspondingly decreasing the number of KRGAI-PLANT layers, we investigate how the initial number of GNN layers influences overall model performance.

**Figure 9:**
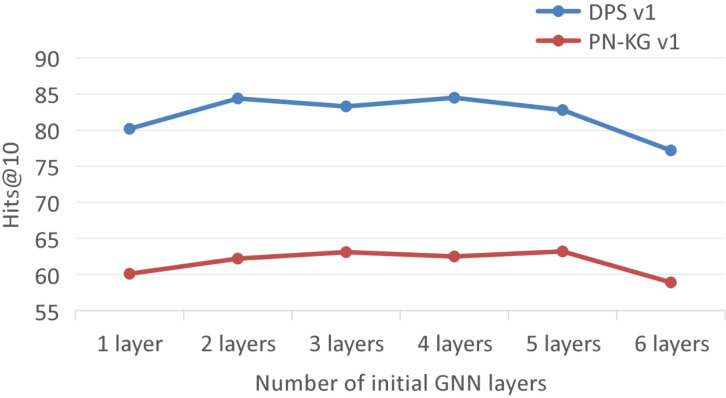
Impact of the initial number of GNN layers on model performance.

**Figure 9** illustrates the variation in model performance, measured by Hits@10, under different initial numbers of GNN layers. It can be observed that model performance initially improves as the number of initial GNN layers increases. Subsequently, further increasing the initial GNN layers while proportionally reducing the number of KRGAI-PLANT layers has no substantial impact on overall performance. Specifically, on the DPS-v1 dataset, performance peaks when the initial number of GNN layers is 2, whereas on the PN-KG dataset, the maximum performance is achieved with 3 initial GNN layers. However, when the initial number of GNN layers reaches 6, model performance declines significantly. Based on this analysis, the following conclusions can be drawn:

1. The experiments demonstrate that model performance improves to a certain extent with an increasing number of initial GNN layers. This validates the effectiveness of the two-stage feature extraction process in the GNNs-KRGAI-PLANT framework and suggests that performing global information propagation on initial node features, which primarily contain query-specific information, yields limited benefits.
2. When the initial number of GNN layers reaches 6, model performance drops considerably. In this configuration, the KRGAI-PLANT modules are entirely removed, and the model loses the ability to perform global information interaction, leading to the observed performance degradation. These results robustly confirm the efficacy of the global information module, aligning with the findings from the ablation studies.

#### 4.7.3 Impact of feature fusion methods on model performance

**Figure 10:**
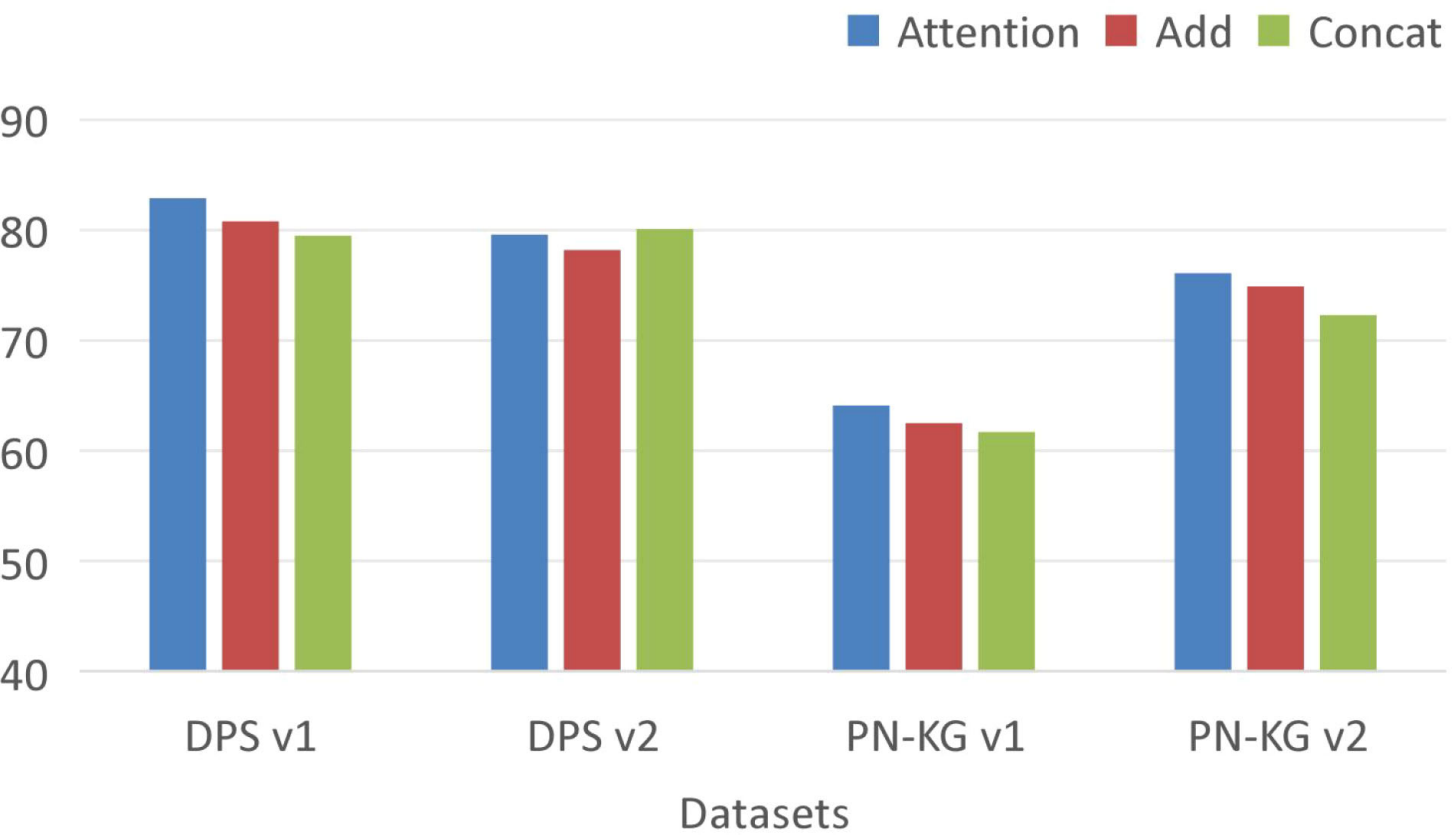
Impact of feature fusion methods on model performance.

In this subsection, we conduct experiments to investigate the impact of the feature fusion method discussed in Section 3.2 on model performance. In our experiments, we evaluate on four datasets by removing the attention-based feature fusion and replacing it with either simple addition or concatenation, thereby exploring how different fusion strategies affect performance. As shown in **Figure 10**, across all four datasets, attention-based feature fusion consistently demonstrates a clear advantage over the other fusion approaches. The underlying reason is that different entities rely on distinct types of information: some nodes prioritize local topological structure, while others require more global information to address long-range dependencies. The attention mechanism enables each entity to automatically select the most relevant information, thereby enhancing the overall performance of the model.

## 5 Conclusions

This study addresses the challenge of long-distance relationship reasoning in plant knowledge graphs by proposing the KRGAI-PLANT model. Through a synergistic dual-channel architecture integrating global and local information pathways, the model effectively overcomes inherent limitations of traditional graph neural networks in modeling multi-hop dependencies. The global module employs a linear attention mechanism to enable direct interaction among all nodes across the graph, thereby overcoming distance constraints in information propagation. The local module focuses on capturing entity neighborhood structures, providing fundamental support for reasoning. An adaptive fusion mechanism dynamically balances the importance of different information sources. Experimental results demonstrate that KRGAI-PLANT exhibits superior performance in long-path reasoning tasks on plant knowledge graphs, significantly outperforming existing methods, particularly in mining complex associations of four or more hops. By effectively capturing the hierarchical and networked nature of botanical knowledge, the model shows promising application potential across various domains, including taxonomy, ecology, and medicinal botany. Interpretability analysis via attention weights further enhances the model’s credibility and offers novel insights into understanding complex biological relationships. A limitation of this study is that the interpretability analysis remains preliminary; a systematic evaluation of attention patterns against biological ground truth is left for future work.

Looking ahead, the KRGAI-PLANT framework holds substantial potential for further development in several specific directions, which will help strengthen its functional capabilities and expand its practical value in real-world scenarios.

First, multimodal knowledge fusion represents a key area for advancement. While this study primarily relies on symbolic triple-based knowledge, plant science is rich in multimodal data—ranging from plant images and genomic sequences to hyperspectral sensor data. A viable approach to enhance the framework is to incorporate visual features, extracted via Convolutional Neural Networks (CNNs) or Vision Transformers (ViTs), as node attributes within the knowledge graph. For example, embedding leaf shape characteristics can enrich morphological entities, enabling the model to synthesize both symbolic relational data and visual similarity information for more comprehensive reasoning. In the same vein, gene sequence embeddings—such as those generated by DNABERT and other genomic language models—can be integrated with taxonomic entities, facilitating cross-species comparative analysis and more accurate trait prediction.

Second, integrating temporal reasoning capabilities to address dynamic processes is essential. Plant growth, phenological cycles, and ecological succession are all inherently time-dependent phenomena. Extending KRGAI-PLANT to support temporal knowledge graphs—where edges are labeled with timestamps or valid time windows—will enable the framework to reason about dynamic processes, such as crop rotation cycles and the spread of pest outbreaks. This can be accomplished by introducing temporal attention mechanisms or adapting state-of-the-art temporal KG reasoning models (e.g., TGN, RE-NET) into the existing dual-channel architecture, allowing the model to capture and learn how relationships evolve over time.

Third, scalable deployment is critical to translating this research into practical agricultural tools. To bridge the gap between academic research and on-the-ground application, future work should prioritize model compression and edge deployment. Lightweight versions of KRGAI-PLANT can be optimized to enable real-time inference on resource-limited devices, such as drones and smart sensors, supporting on-site decision-making for tasks like crop disease detection and irrigation scheduling. Additionally, integrating the framework with large language models (LLMs) can enhance human-machine interaction: farmers will be able to query the system using natural language (e.g., “What is the risk of powdery mildew in my wheat field next week?”) and receive interpretable, knowledge-based responses.

Fourth, toward systematic interpretability evaluation, we plan to adopt Functional Semantic Activation Mapping (FSAM) (Raj and Mileo, 2026) in future work. Unlike local explanation methods, FSAM provides a global, layer-wise view of semantic representations in GNNs. A key insight from their work is that higher accuracy does not necessarily imply more faithful semantic understanding—models may make correct predictions for incorrect reasons. Applying FSAM to KRGAI-PLANT could help us validate whether its performance gains stem from genuine semantic reasoning, quantify how semantic coherence changes across layers, and determine the optimal depth before semantic collapse occurs. This would transform our interpretability claims from qualitative intuition into quantitative validation. By advancing these three directions, the KRGAI-PLANT framework can evolve from a static reasoning tool into a dynamic, multimodal, and temporally sensitive intelligent system. This transformation will lay a solid foundation for its application in next-generation precision agriculture and ecological monitoring, unlocking new possibilities for sustainable agricultural development.

## Data Availability

The raw data supporting the conclusions of this article will be made available by the authors, without undue reservation.
